# Use of RENAL Nephrometry Scores for Predicting Tumor Upgrading Between Core Biopsies and Surgical Specimens: A Prospective Ex Vivo Study

**DOI:** 10.1097/MD.0000000000000581

**Published:** 2015-02-27

**Authors:** Gui-Ming Zhang, Yao Zhu, Hua-Lei Gan, Hong-Kai Wang, Guo-Hai Shi, Hai-Liang Zhang, Bo Dai, Chao-Fu Wang, Ding-Wei Ye

**Affiliations:** From the Department of Urology (GMZ, YZ, HKW, GHS, HLZ, BD, DWY); Department of Pathology (HLG, CFW, Fudan University Shanghai Cancer Center; and Department of Oncology (GMZ, YZ, DWY), Shanghai Medical College, Fudan University, Shanghai, China.

## Abstract

Determination of Fuhrman grade (FG) on biopsies of renal masses is relatively inaccurate, being prone to underestimating the true grade as ascertained from surgical specimens. This study evaluated whether anatomical features of tumors could predict tumor upgrading between core biopsies and surgical specimens.

We prospectively enrolled 249 patients undergoing surgical resection of solid renal masses at our institution from 2012 to 2013. Tumor anatomical features were defined using RENAL nephrometry scores. Two peripheral and 1 central ex vivo core biopsies were taken from surgical specimens with an F18-gauge needle. Logistic regression was used to assess associations between covariates and FG upgrading. A comprehensive nomogram was constructed to quantitate the probability of tumor upgrading.

The median tumor size was 4.75 cm and FG upgrading occurred in 43.6% of cases. In tumors of low, intermediate, and high complexity, the risk of FG upgrading was 22.0%, 47.6%, and 50.6%, respectively. According to multivariate analyses, anatomical features R (radius) and L (location) scores correlated significantly with FG upgrading. A combination of anatomical features and core biopsy findings predicted tumor upgrading with an accuracy of 0.884. With a threshold of 30%, our nomogram identified 92.4% of cases with upgrading; however, it overrated 26.8% of patients without upgrading.

This ex vivo prospective study demonstrated that RENAL nephrometry score can aid prediction of FG upgrading between core biopsies and surgical specimens. Our nomogram uses anatomical features to predict true FG from renal biopsies.

## Abstract 2

Supplemental Digital Content is available in the text

## INTRODUCTION

With advances in imaging modalities, the incidence of asymptomatic renal masses has risen dramatically, posing therapeutic dilemmas that did not previously exist.^1^ Urologists are now faced with increasing numbers of small renal masses that are more likely to be benign,^2–4^ or biologically less aggressive if malignant, than larger renal tumors. Now that other options besides extirpative surgery, such as active surveillance or thermal ablation, are available for patients with small renal masses, especially those who are poor surgical candidates, accurate pathological information is pivotal to accurate stratification of patients into risk categories. In addition, selection of appropriate therapeutic strategies for patients with inflammatory lesions, lymphoma, or metastases in the kidneys depends on reliable pathological evidence. Therefore, increasing numbers of renal mass biopsies (RMBs) have been performed over the past few decades.^5,6^ Although the rates of complications and tumor seeding are reportedly lower than suspected, the use of RMBs is still controversial because of the high incidence of inconclusive results.

Improvements in pathological techniques have increased the accuracy of discriminating between benign and malignant lesions, whereas differentiating indolent from aggressive renal tumor remains unreliable. Provided the sample is adequate, according to most recent studies, RMB is 84% to 96% accurate at distinguishing benign from malignant masses.^7–9^ In contrast, these studies report that the accuracy of Fuhrman grade (FG) is as low as 70%.^8^ However, FG remains an important prognostic indicator for guiding clinical decision making.^10^ Even for patients with advanced or metastatic renal cell carcinomas (RCCs), accurate information of FG may be used to evaluate disease aggressiveness and prognosis, and guide treatment strategies, including whether to proceed with neoadjuvant targeted therapy or cytoreductive nephrectomy (CN). Failure to assess FG may lead to under or overestimation of likely outcome, resulting in under or overtreatment, respectively; the former in particular has undesirable consequences. Hence, besides determining whether a lesion is malignant, acquisition of precise information for determining the FG accurately is also of concern.

Since it was proposed in 2009, the RENAL nephrometry score (RNS) has used anatomical features to aid preoperative prediction of the character of renal masses.^11^ Several studies have evaluated the feasibility and accuracy of RNS using external validation and have reported that it is a reproducible and helpful tool in clinical practice.^12–14^ A recent study has also suggested that RNS may be associated with FG.^13^ However, whether RNS provides additional information in the setting of RMBs remains an unanswered question. To test this hypothesis, we enrolled consecutive patients with renal masses in this prospective study and performed ex vivo biopsies of surgical specimens to evaluate the role of RNS in predicting FG upgrading between core biopsies and surgical specimens.

## METHODS

### Patients and Mimicked RMB

This study included 249 consecutive patients with renal masses who underwent renal surgery (open or laparoscopic radical, partial, or palliative nephrectomy) at Fudan University Shanghai Cancer Center, Shanghai, China, from January 2012 to June 2013. Two senior urologists who were blinded to the pathological information independently reviewed the patients’ computed tomography or magnetic resonance images and assigned scores for the 6 components: R, radius; E, exophytic/endophytic properties; N, nearness to collecting system or sinus; A, anterior/posterior; L, location relative to polar lines; and H, hilar (tumor touching main renal artery or vein) of the RNS.^11^ The patients were divided into the following 3 groups according to their total RNS: low risk (4–6), intermediate risk (7–9), and high risk (10–12). Data on age, sex, body mass index (BMI), smoking status, and history of hypertension and diabetes were obtained from electronic medical records.

RMBs were mimicked by taking 3 ex vivo core biopsies from surgically resected specimens with an 18-gauge needle; two of the cores being obtained from the peripheral part of the tumor and the third from the central part. The core biopsies and surgical specimens were assessed independently according to the WHO 2004 FG classification system by an experienced genitourinary pathologist. If the biopsies were insufficient to confirm histological diagnoses or determine FG, they were considered noninformative and these patients were classified as having tumor upgrading in the subsequent analyses.

Written informed consent was obtained from all patients before participation, and the study protocol was approved by the Institutional Research Review Board of the Fudan University Shanghai Cancer Center.

### Statistical Analysis

Differences in categorical variables were compared using Pearson χ^2^ test. Logistic regression was used to determine odds ratio (OR) and 95% confidence interval (CI) of covariates. Receiver-operating characteristic curve and area under the curve (AUC) were used to determine the efficacy of the predictive variables. A nomogram was constructed to provide optimal graphic models for quantitating probabilities. *P* values were 2 sided and *P* < 0.05 was considered statistically significant. Statistical analyses were carried out using SPSS version 20.0 (IBM Corporation, Somers, NY) and R 2.13.0.

## RESULTS

### Clinical Characteristics

Ex vivo core biopsies of renal tumors from 249 consecutive patients were obtained. FG is of prognostic value only in clear cell and papillary RCC; therefore, 45 patients with other pathological diagnoses were excluded, including 13 with angiomyolipoma, 3 with oncocytoma, 11 with chromophobe RCC, 5 with collecting duct carcinoma, 3 with Xp11.2 translocation RCC, 3 with urothelial carcinoma, and 7 with renal sarcoma. Among the included patients, 190 had clear cell RCC and 14 papillary RCC. The median patient age was 54 years (range 15–82 years) with a male predominance (64.2%). The median tumor size was 4.75 cm (range 1.0–20.0 cm), 70 of the masses (34.3%) being ≤4 cm. The RCCs were removed by radical nephrectomy in 121, partial nephrectomy in 70, and palliative nephrectomy in 13 cases.

Ex vivo core biopsies of the renal tumors yielded noninformative results in 15 cases, including nonmalignant tissue in 5 cases, and insufficient samples to determine the grade in 10 cases. Pathological examination of the surgical specimens showed grade 1 in 8, grade 2 in 93, grade 3 in 90, and grade 4 in 13 cases. When findings on core biopsies and surgical specimens were compared, the FG was upgraded in 89 tumors (43.6%). The probability of upgrading was significantly greater for tumors >4 cm than for those ≤4 cm (47.0% vs 37.1%). Possible associations between tumor upgrading and the clinical characteristics of age, sex, BMI, smoking status, hypertension, diabetes, and pathological subtype were investigated; no significant associations were identified (Table 1).

**TABLE 1:**
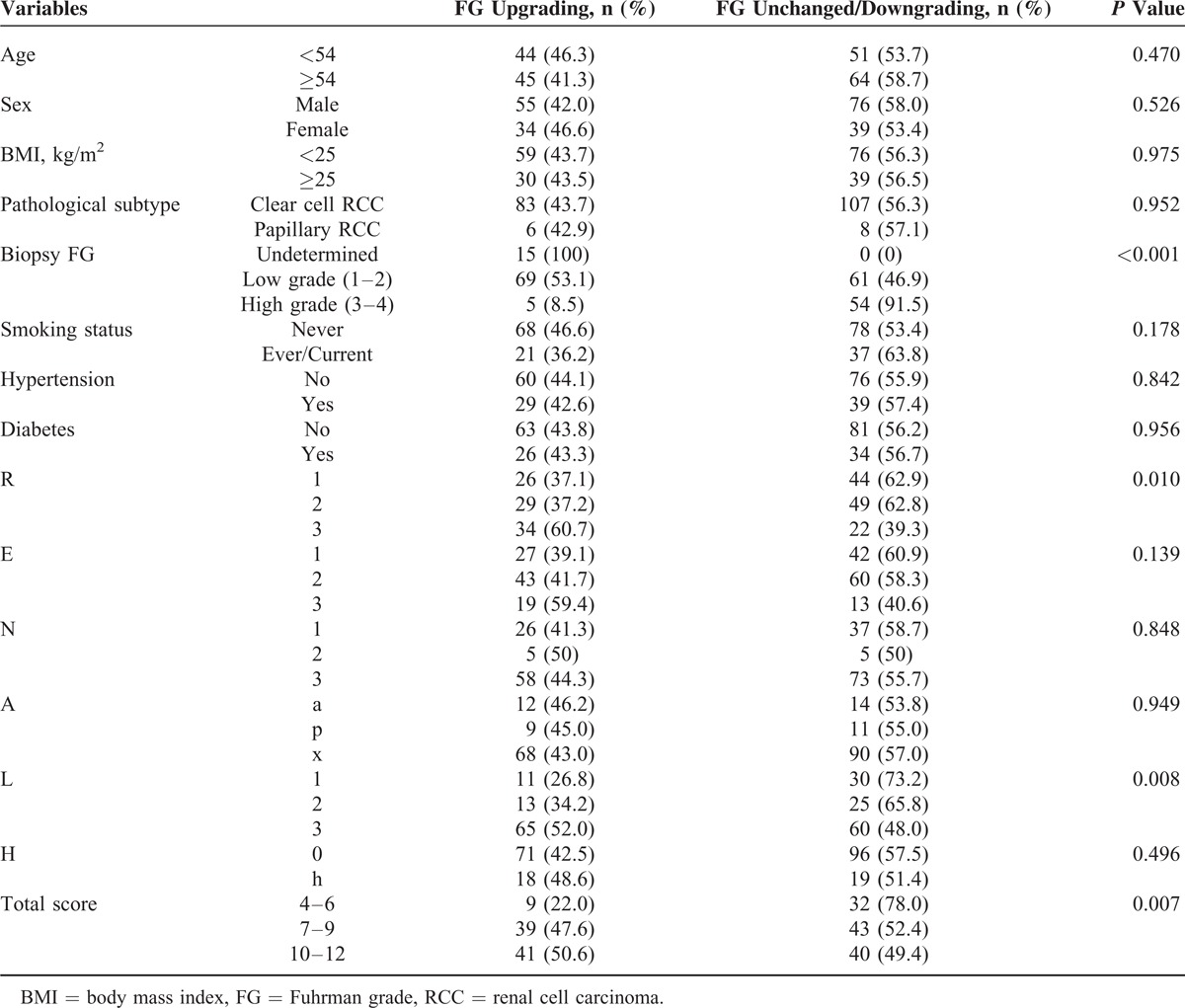
Clinical, Pathological, and Anatomical Features in 204 Patients With RCC

### Associations Between RNS and Tumor Upgrading

Table 1 shows the percentage of FG upgrading according to clinical characteristics, anatomical features, and total RNS. R and L scores were significantly associated with tumor upgrading. Furthermore, total scores, which reflect tumor complexity, were also strongly associated with an increase in FG. Upgrading was over twice as likely in the high-risk as in the low-risk group (50.6% vs 22.0%).

Multivariable logistic regression was used to evaluate the adjusted associations between anatomical features and tumor upgrading. As shown in Table 2, after adjustment for age, sex, BMI, smoking status, hypertension, diabetes, pathological subtype, and biopsy FG, significant associations between FG upgrading and total RNS were observed in both intermediate-risk (OR: 3.009, 95% CI: 1.144–7.916, *P* = 0.026) and high-risk (OR: 4.243, 95% CI: 1.568–11.481, *P* = 0.004) groups. Accounting for the influence of covariates, larger tumor size, tumor location to polar line, and general anatomical complexity were significant indicators of tumor upgrading.

**TABLE 2:**
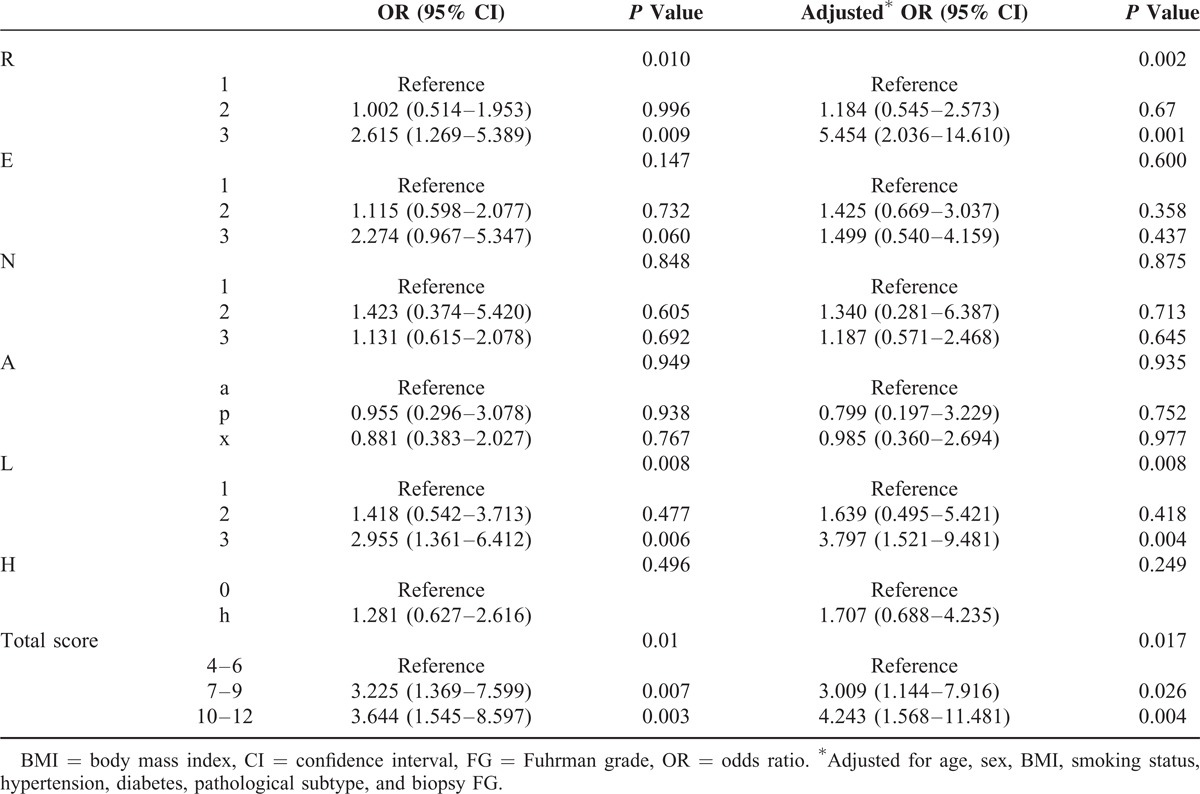
Logistic Regression Analysis of the Associations Between Anatomical Features of Renal Tumors and FG Upgrading

Next we analyzed the association of L and total scores with FG upgrading stratified by tumor size (≤4 or >4 cm). As indicated in Table 3, L score was significantly associated with tumor upgrading, in both small renal masses (≤4 cm) and nonsmall renal masses (>4 cm). However, total score was a significant indicator of FG upgrading only for small renal masses. Marginal significant association was observed in tumors >4 cm.

**TABLE 3:**
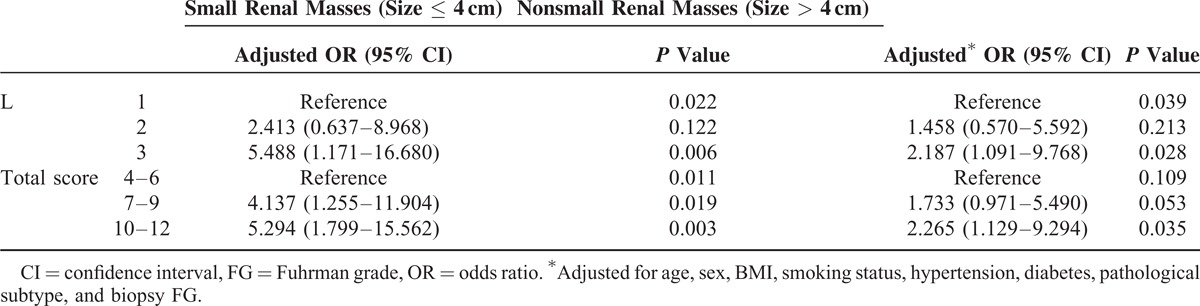
Stratification Analysis for Association Between Anatomical Features of Renal Tumors and FG Upgrading

### Combination of Anatomical Features and Core Biopsies for Predicting Tumor Upgrading

**FIGURE 1:**
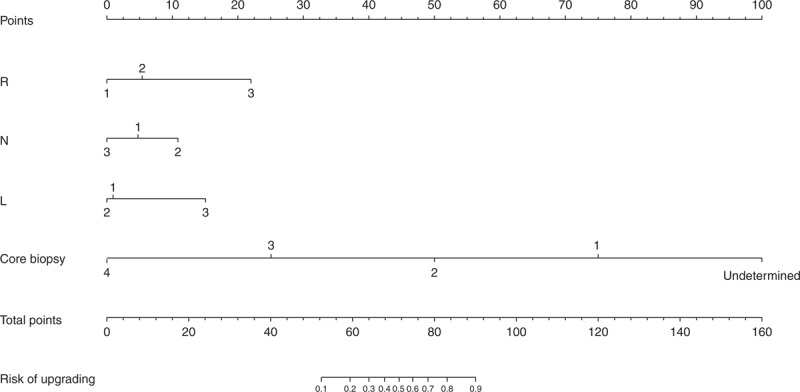
Nomogram for predicting the probability of tumor upgrading in patients with RCC undergoing core biopsy. RCC = renal cell carcinoma.

To achieve the goal of predicting FG upgrading, a multivariate model, which included clinical characteristics, individual anatomical features, and core biopsy results, was constructed. Using backward variable selection based on the “Akaike information criterion,” R, N, and L scores and biopsy grade remained in the final model. The predictive accuracy of the final model was 0.884 (0.841–0.928). The performance of the model in our patients was assessed according to a set of probability thresholds (Table 4 and Supplementary Figure, http://links.lww.com/MD/A219). For example, the nomogram (Figure 1) correctly identified tumor upgrading in 92.4% of patients with a predicted probability of tumor upgrading of ≥0.3 (AUC = 0.723), while overrating 26.8% of patients without upgrading.

**TABLE 4:**
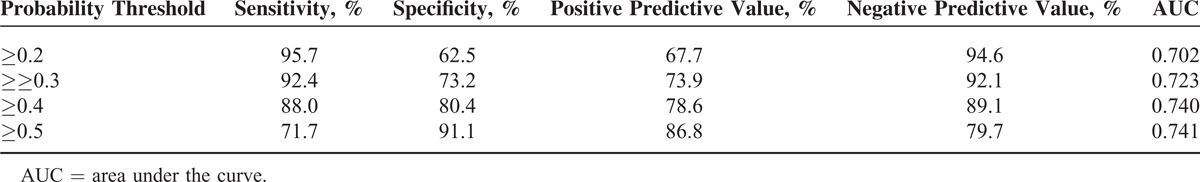
Prediction Performance of the Nomogram According to Various Probability Thresholds

## DISCUSSION

In addition to patient factors and surgical expertise, the choice of treatment algorithm for renal masses is mainly based on evaluating their biological potential. Consequently, clear identification of prognostic factors would help urologists distinguish benign from malignant renal lesions, as well as from progressive malignancies that require immediate intervention, and indolent ones for which active surveillance or ablation may be appropriate. With the advent of targeted agents, expanded therapeutic options or combinations are available for patients with advanced RCC or with evidence of metastatic disease. For these patients, accurate determination of malignant potential may likewise provide useful information for prognosis estimation and treatment planning. For example, appropriate clear cell RCC subtype assessment is important before enrolment into preoperative clinical trials.^15^ Karam et al^16^ showed that patients characterized by a high prevalence of low-grade disease had a high probability of response after neoadjuvant axitinib treatment. High FG or sarcomatoid differentiation is a poor prognostic evidence and might show a lack of survival benefit from CN.^17^

With advances in techniques, notable improvements in RMB for diagnosis of RCC have been made. Lane et al^9^ reported an average diagnostic accuracy of 96% among major clinical studies conducted from 2001 to 2008. However, accurate determination of FG, which characterizes the biological potential of RCCs and carries immense prognostic significance, is difficult because of the heterogeneity of RCCs and issues associated with incomplete sampling.^18^ Blumenfeld et al^19^ reported that RMB underestimates FG in a significant proportion of biopsies. In their series, subsequent FG upgrading occurred in 55% of cases, whereas overestimation of FG occurred in only 1 case. Another study group reported a comparable finding of 52% accuracy in identifying FG.^20^ Abel et al^17^ assessed the accuracy of RMB in 104 metastatic RCC patients before undergoing CN, and found that only 33 (31.7%) had the same FG with the nephrectomy specimen. With respect to FG of 4, only 24.6% were accurately identified.^17^ Similar to previous studies, underestimation of FG occurred in 43.6% of cases in our study. In a retrospective study that aimed to investigate the accuracy of determining the management of small renal masses through RMB, 8.3% (11/133) of patients were incorrectly managed.^21^ It is noteworthy that 81.8% (9/11) of instances of incorrect management were ascribed to FG upgrading. Therefore, FG upgrading commonly affects clinical decision making, which deserves full consideration.

Pretreatment histological confirmation substantially aids the estimation of prognosis and various endpoints and informs patients about treatment options. Therefore, attempts have been made to predict the pathological features of renal masses based on their anatomical characteristics. Kutikov et al^12^ were the first to examine the ability of various anatomical features of renal masses to predict malignant and high-grade disease.^11^ Several case series have sought to validate externally their concept, with inconsistent results. In a cohort of patients with T1a disease, Fujita et al^22^ reported a positive association between E score 1 and benign lesions. Wang et al^13^ confirmed the ability of RNS to predict high-grade RCC in an independent cohort. Analogous results were reported by Satasivam et al^23^ who found that RNS is positively associated with tumor aggressiveness. Tay et al^24^ have demonstrated that high RNS is associated with pathological upstaging of clinical T1 RCCs, and that R and L scores are independent predictors of this upstaging. However, another study found that the RNS nomogram was inferior at predicting high-grade RCC, despite having a comparable ability to predict malignancy.^25^ Our study used ex vivo core biopsies to move a step forward in the application of RNS in clinical practice. After adjustment for confounding factors, we found that 2 tumor anatomical features (R and L) and total RNS were significantly associated with FG upgrading. Furthermore, RNS and risk of FG upgrading tended to increase in parallel. It should be noted that tumors of intermediate or high complexity were at least 3 times more likely to be upgraded than low-risk tumors. Ablative treatment rather than partial nephrectomy should be considered for complex tumors, therefore, low FGs in preablative biopsies should be interpreted with caution. The nomogram we have developed has good sensitivity for identifying patients at risk of upgrading at predefined probability thresholds. Therefore, this prediction tool may aid in decision making concerning treatment and subsequent follow-up.

We performed additional analyses to investigate whether the prognostic value of anatomical features was modified by tumor size, and found that both high L score and high total score remained significant predictors in large tumors. The subgroup analyses indicated that the predictive performance of RNS was homogeneous for different tumor size. Therefore, we included large tumor size in the final analysis to increase sample size for model construction.

Why anatomical features help in predicting tumor upgrading is still unknown. We speculate that they may, in part, explain aggressiveness and heterogeneity of RCCs. Tumor size, represented by R score, is positively associated with tumor grade.^3,26^ Core biopsies from large renal masses are more likely to provide inadequate samples. A close relationship between L score and tumor grade was confirmed in our study and that of Kutikov et al^12^; however, the precise mechanism has not been identified. It is particularly interesting that FG upgrading occurred in only 26.8% (11/41) of patients with tumors confined to 1 renal pole in our study. E scores also showed a tendency toward being associated with upgrading of infiltrative tumors; thus, we postulate that tumors that have invaded less of the renal parenchyma may be less aggressive. Urologists should recognize the high possibility of FG upgrading between RMB and surgical specimens in tumors with high RNS, especially those that are large and located close to the polar line.

We acknowledge that our study had several limitations. First, ex vivo biopsies were only partially comparable with preoperative biopsies obtained in clinical practice; however, few studies have compared findings of percutaneous preoperative RMB samples with those of surgical specimens in 100% of cases. Second, all the patients enrolled in this study were treated at a single institution, which may have resulted in selection bias. However, our use of RNS of ex vivo RMB samples was a step forward compared with recent attempts at validation by retrospective imaging. Multivariate analyses demonstrated the independent predictive values of R and L individually and RNS as a multifactorial score. Therefore, RNS may improve the interpretation and predictive value of RMBs. Our findings require validation with larger cohorts.

In summary, our results suggest that RNS is a useful tool in predicting FG upgrading of RCCs. The nomogram that we constructed may reduce misclassification of tumor grade in RMBs and thus improve clinical decision making. Our findings require evaluation in larger cohorts.
